# Securing Internet-of-Medical-Things networks using cancellable ECG recognition

**DOI:** 10.1038/s41598-024-54830-2

**Published:** 2024-05-13

**Authors:** Samia A. El-Moneim Kabel, Ghada M. El-Banby, Lamiaa A. Abou Elazm, Walid El-Shafai, Nirmeen A. El-Bahnasawy, Fathi E. Abd El-Samie, Atef Abou Elazm, Ali I. Siam, Mohamed A. Abdelhamed

**Affiliations:** 1 Tanta High Institute of Engineering and Technology (THIET), Tanta, Egypt; 2 Department of Industrial Electronics and Control Engineering, Faculty of Electronic Engineering, Menoufia University, Menouf, 32952 Egypt; 3 Department of Microelectronics, Electronics Research Institute, Nozha, Egypt; 4 Department of Electronics and Electrical Communications Engineering, Faculty of Electronic Engineering, Menoufia University, Menouf, 32952 Egypt; 5 Department of Computer Science and Engineering, Faculty of Electronic Engineering, Menoufia University, Menouf, 32952 Egypt; 6 Department of Embedded Network Systems Technology, Faculty of Artificial Intelligence, Kafrelsheikh University, Kafr El-Shaikh, Egypt; 7 Department of Communications and Computers Engineering, Higher Institute of Engineering, El Shorouk Academy, El Shorouk City, 11837 Egypt

**Keywords:** IoMT, ECG signals, Cancellable biometrics, Signal separation, Health care, Engineering

## Abstract

Reinforcement of the Internet of Medical Things (IoMT) network security has become extremely significant as these networks enable both patients and healthcare providers to communicate with each other by exchanging medical signals, data, and vital reports in a safe way. To ensure the safe transmission of sensitive information, robust and secure access mechanisms are paramount. Vulnerabilities in these networks, particularly at the access points, could expose patients to significant risks. Among the possible security measures, biometric authentication is becoming a more feasible choice, with a focus on leveraging regularly-monitored biomedical signals like Electrocardiogram (ECG) signals due to their unique characteristics. A notable challenge within all biometric authentication systems is the risk of losing original biometric traits, if hackers successfully compromise the biometric template storage space. Current research endorses replacement of the original biometrics used in access control with cancellable templates. These are produced using encryption or non-invertible transformation, which improves security by enabling the biometric templates to be changed in case an unwanted access is detected. This study presents a comprehensive framework for ECG-based recognition with cancellable templates. This framework may be used for accessing IoMT networks. An innovative methodology is introduced through non-invertible modification of ECG signals using blind signal separation and lightweight encryption. The basic idea here depends on the assumption that if the ECG signal and an auxiliary audio signal for the same person are subjected to a separation algorithm, the algorithm will yield two uncorrelated components through the minimization of a correlation cost function. Hence, the obtained outputs from the separation algorithm will be distorted versions of the ECG as well as the audio signals. The distorted versions of the ECG signals can be treated with a lightweight encryption stage and used as cancellable templates. Security enhancement is achieved through the utilization of the lightweight encryption stage based on a user-specific pattern and XOR operation, thereby reducing the processing burden associated with conventional encryption methods. The proposed framework efficacy is demonstrated through its application on the ECG-ID and MIT-BIH datasets, yielding promising results. The experimental evaluation reveals an Equal Error Rate (EER) of 0.134 on the ECG-ID dataset and 0.4 on the MIT-BIH dataset, alongside an exceptionally large Area under the Receiver Operating Characteristic curve (AROC) of 99.96% for both datasets. These results underscore the framework potential in securing IoMT networks through cancellable biometrics, offering a hybrid security model that combines the strengths of non-invertible transformations and lightweight encryption.

## Introduction

Over the past ten years, there has been a revolution on the Internet of Things (IoT) due to developments in networking technologies and protocols. Governments and communities approved  IoT applications, because they give users opportunities to have control over their peripherals and assets. The concept of IoT has been expanded to the healthcare industry with this development. To follow the state of individuals suffering from long-term diseases, new networks known as IoMT have been launched^1,2^. These IoMT networks should be connected to patients and caregivers in order to facilitate routine biomedical measurement exchange and remote access to testing and diagnosis.

Because IoMT networks are susceptible to a wide range of attacks, their security is a vast area of research. During data transmission over IoMT networks, attacks could involve anything from fake users to fake nodes^3,4^. Furthermore, in ways akin to SQL injection, attackers might be able to breach IoMT networks and manipulate patient data^1,5^. It is obvious that such actions could endanger the patients' lives, since they will result in inaccurate diagnosis and course of treatment. Answering the query “How is the network accessed by the patients??” is the first step towards ensuring the security of IoMT networks. Patients have the option to base their IoMT network access on biometric-based authentication, according to Xin et al.^6^. For the patients to access the IoMT networks, they could use a framework that is based on the combination of facial, fingerprint, and finger vein features.

The IoMT network primary goal is to monitor patient conditions associated with chronic diseases. Consequently, it is advised to use one of the biometrics associated with the ongoing patient measurements in the access process. The ECG is highly recommended for this task. The heart electrical activity is measured with the ECG^7–9^. Through a combination of parasympathetic and sympathetic mechanisms, the ECG waveform is regulated by the autonomic nervous system. As a result, each instance is unique for each subject. Therefore, ECG signals can be utilized for authentication^10,11^. With ECG signals used as biometrics, patients will not be required to provide additional biometrics for authentication, and hence the ECG signals are better suited for use in IoMT networks.

To reduce the effects of attacks, many contemporary access control system designers, particularly in the medical sector, place a strong emphasis on biometric authentication in place of passwords, credit cards, or token-based verification systems. Biometric-based authentication systems' ease of use is thought to be beneficial in many important applications. Biometric traits, like voice^12^, electroencephalography (EEG), photoplethysmography (PPG)^13^, ECG, face, hand geometry^14^, and ear shape, are distinct for each user, and cannot be replicated. Users can therefore use them with ease in remote access systems. ECG scanning provides the advantage of continuously enabling ECG signals for patients in the context of patient monitoring. As a result, even in situations of fatigue and lack of capacity to provide additional biometric traits, patients can simply rely on ECG signals to connect to the healthcare system. Unfortunately, one of the weakest points in the biometric system is the biometric acquisition, which makes the biometric trait vulnerable to theft and other forms of attacks. It is important to secure the primary biometrics used during the access process to stop attackers from pretending to be other users.

ECG signals adhere to the two primary prerequisites for use of human biometrics in authentication systems: universality and permanence. Given that the ECG signals of all subjects can be continuously monitored, universality is preserved. High permanence of ECG-based authentication systems is guaranteed by the signals’ long-term invariance. Furthermore, with ECG signals, there is a continuous guarantee for aliveness detection. ECG signals can be used for biometric authentication over IoMT networks due to all of these features.

Regretfully, databases must hold biometric features or attributes in order for biometric-based authentication systems to operate, effectively. Attacks can occur at any point in a biometric system, from biometric acquisition to decision-making^15^. For this reason, cancellable biometrics have become more popular. Users can use alternative biometric templates made with non-invertible transformations or encryption schemes by creating new cancellable biometric templates. The purpose of cancellable biometrics is to safeguard the original biometric information, while keeping the ability to discriminate between users^16^.

After an exhaustive review of the existing cancellable biometric systems, we found that encryption-based systems are vulnerable to record multiplicity attacks, and non-invertible transform-based systems are susceptible to brute-force attacks^3,15^. For brute-force attacks to be prevented, strong encryption techniques with very long keys are required. They might not be appropriate for high-speed biomedical applications. Introducing a hybrid framework for cancellable biometrics that combines a non-invertible transform and a lightweight encryption scheme is a sophisticated solution to this problem. With this approach, we can achieve high authentication accuracy, high speed of operation, and high privacy of users by cascading these two stages.

It is clear that IoMT applications are developing right now. Resilient and efficient access mechanisms are necessary for them. It is not advised to gain access to these applications using raw biometrics, since they are susceptible to hacking attempts. Thus, there is presently a dearth of research into the development of cancellable biometric recognition systems that are particularly well-suited for use in IoMT applications. In the context of IoMT, cancellable biometrics and encryption-based algorithms have not yet been thoroughly investigated, despite being extensively studied in other contexts. As a result, there is a chance that the biometric recognition technologies currently in use in IoMT applications are insufficiently secure to stop misuse and unauthorized access to personal health data. Conventional biometric systems are susceptible to breaches of security and invasions of privacy, because biometric data is kept in a central database. Consequently, a safe and private biometric recognition system is required, one that can be reliably used for authentication, while safeguarding patient data. The proposed cancellable biometric recognition framework is a good candidate to address this problem. It is based on lightweight encryption and a signal separation algorithm to induce distortion in the original ECG signals. However, more investigation is required to confirm the system performance and pinpoint its potential restrictions or disadvantages as well. The efficacy and generalizability of this system have not yet been thoroughly assessed.

For patients, it makes sense to benefit from cancellable biometrics’ recent growth in IoMT applications for access control. For patients to deal with IoMT applications, the best biometric traits are the ECG signals, which are continuously monitored. The two main tools for the development of cancellable biometric systems, namely non-invertible transforms and biometric encryption, are not adequate on their own, because they are susceptible to specific kinds of attacks. Combining them can raise the cancellable templates' level of security, as this paper reveals. Avoiding excessive complexity in the combination process is an essential requirement that must be taken into account. For this reason, lightweight encryption is used, and a signal separation algorithm is implemented as a non-invertible transform. The rationale behind the utilization of signal separation is its ability to give two low-correlation signal components from two signals having some sort of correlation. As a result, if we begin operation with an ECG signal and an auxiliary audio signal for the same person, with some sort of correlation of any level, the two resultant signals after separation will be of minimal correlation. In other words, the two resultant signals after separation can be considered as distorted signals that depend in their origin on the ECG signal used. One of these distorted signals can be used as a cancellable template for the user. The combination of lightweight encryption and blind signal separation improves security effectiveness of original cancellable templates.

Consequently, the motivations behind this work are:

- To create a more secure biometric recognition framework for IoMT applications. Cancellable biometric systems generate a new biometric template for every authentication request, thereby mitigating the vulnerability of traditional biometric systems to attacks.
- To preserve patients' private information. Biometric data is further safeguarded by cancellable biometric systems, which ensure that biometric data cannot be reverse engineered to reveal the original biometric features.
- To improve the authentication efficacy in IoMT applications. Traditional biometric systems require a centralized database storage of biometric data with certain precautions to safeguard original biometrics , which may be expensive and time-consuming to maintain. In contrast, cancellable biometric systems do not require these precautions, because it is possible to create new templates in case of compromise.
- To investigate the possibility of using lightweight encryption in conjunction with a 2 × 2 blind signal separation module for cancellable biometric recognition. Although this framework is thoroughly studied in the context of IoMT applications, it may be adopted on a large scale.
- To develop a framework that can be applied to real-world IoMT applications, such as remote health monitoring and patient identification.

This paper presents a cancellable ECG recognition framework that starts at the ECG acquisition stage. In order to produce a non-invertible dynamic range modification in the ECG signals, a 2 × 2 blind signal separation module is applied to each ECG biometric signal  with an audio signal in order to obtain two distorted outputs with minimal correlation. This process leads to templates with distortions that cannot be reverted. Next, a straightforward XOR encryption step is applied using a key that is unique to each patient. This step raises the users’ degree of privacy. Every user has an easy way to choose his key. Furthermore, his original ECG biometric is not saved in the system database. The user can quickly alter the key he has chosen or the audio signal that the separation algorithm begins with in case of compromise.

This paper main contribution is a trustworthy framework for authentication in addition to ensuring aliveness of patients in IoMT networks. By creating cancellable biometric templates that are non-invertible, the biometric recognition framework will be more resilient to attempts of tampering or theft of original biometrics. Generally, a cancellable biometric system is a system that depends on generating distorted, modified versions of the biometric data in a non-invertible way. There should be no information provided about the actual biometric traits by this one-way transformation. By comparing the new user's transformed or distorted template with the distorted templates kept in the database, the authenticity of the user can be verified.

The main contributions of this work are:

- Providing innovative a cancellable biometric recognition framework based on a 2 × 2 blind signal separation module that is applied to ECG and audio signals, as well as a lightweight encryption algorithm. This framework is recommended for IoMT applications.
- Creating user-specific patterns for lightweight encryption using XOR operation to increase security of biometric traits. The lightweight encryption algorithm is intended to reduce the system processing and storage requirements, making it appropriate for IoMT devices with limited resources.
- By creating cancellable biometric templates that can be used for authentication without subjecting the original biometric data to breaches, the proposed framework provides an excellent degree of security and privacy.
- Several ECG databases are used to assess the suggested framework, and results demonstrate that it maintains high degrees of security and privacy, while achieving high recognition accuracy and low computational cost.

Lastly, the proposed framework is compared to previously-published studies that made use of the same datasets. The results show that the proposed framework performs better in terms of authentication accuracy than other previous counterparts. The main advantage of this work is that the sophisticated signal separation module induces the required level of distortion in cancellable templates without large complexity. Moreover, the lightweight encryption adds to the degree of security of templates, while keeping the high ability to identify users.

The paper is organized as follows. The recent related works are discussed in “Related Work” Section. The proposed cancellable ECG biometric recognition framework is explained in “Proposed Cancellable ECG Recognition Framework” Section. “Experiments” Section presents the simulation results and discussion. “Conclusions and Future Work” Section provides concluding remarks and future research guidelines in this area.

## Related work

Numerous studies on person identification using ECG signals have been published in the literature. An algorithm for person identification based on ECG signals was introduced by Lee and Kwak^17^. Principal component analysis and Eigen value decomposition were the main tools of their work. The robustness of this algorithm to noise effects has been verified. The authors obtained a 98.25% classification accuracy.

An ECG-based identification method based on sparse feature representations was presented by Huang et al.^18^. In an overall optimization framework, users' sparse feature patterns are subjected to similarity tests. In the recognition process, a regularization problem and a set of constraints are considered. The purpose of this method was to provide authentication for access to embedded smartphone applications. Its relative complexity stems from the requirement to solve an optimization problem and perform Eigen decomposition of matrices.

Furthermore, ECG-based identification was presented by Barros et al.^19^ with pre-processing steps before the identification process. In order to concentrate on the most representative ECG features for identification, pre-processing steps, such as outlier removal, QRS complex segmentation, and noise reduction, were carried out on three-second signal segments. Twenty-two features were included in the identification process. Using the PhysioNet Computing in Cardiology 2018 dataset, the authors validated their approach using Random Forest (RF) classifier^20^. On 1500 subjects, this work showed a 92% precision and an 80% accuracy on 1200 subjects.

Finger veins and ECG signals were combined by Su et al.^21^ for human identification. Discriminant Correlation Analysis (DCA) and Canonical Correlation Analysis (CCA) were employed for fusion of the features extracted from each database. This model achieved an EER of 0.144%. This multi-modal approach did better in terms of security and recognition accuracy than the two independent unimodal implementations.

Another methodology for human authentication based on ECG readings from two finger electrodes connected to a smartphone application was presented by Zhang et al.^22^. They chose fiducial feature extraction and used the Discrete Cosine Transform (DCT) to reduce the dimensionality of the features due to its energy compaction property. To evaluate the performance of the model, they employed Support Vector Machines (SVMs) and Neural Networks (NNs). They achieved accuracy levels up to 97.6% and 96.6%, respectively. This model requires 4 s for authentication and 20 s to register a new user.

Hammad et al.^23^ proposed two methods to build an ECG-based cancellable biometric system. They recommended improved matrix manipulation and bio-hashing techniques. Feature vector generation and coding are typically performed with bio-hashing to create irreversible binary codes. However, the matrix manipulation method includes operations like mixing, matrix inversion, row and column permutations, and more. In their work, the authors first extracted the ECG features using the Pan-Tompkins technique, and then utilized an ANN for authentication. Their methods achieved EER values of 0.20 and 0.06.

A cancellable ECG biometric recognition system based on a Generalized Likelihood Ratio Test (GLRT) with randomly-selected hypothesis testing was proposed by Kim et al.^24^. Additionally, they suggested utilization of Guided Filtering (GF) to transform the ECG templates in an irreversible way. Finally, they evaluated the system on the ECG-ID database. It performed better than the conventional Euclidean detector, with a performance index of 94.3%.

An ECG-based human authentication system based on generalized S-transformation and Convolutional Neural Networks (CNNs) was presented by Zhao et al.^25^. To acquire the trajectories of ECG signals in the form of images, the signal segments are first blindly processed with the S-transform. In order to further identify the authorized users, these trajectories are subsequently fed into a CNN as input images. Three distinct databases of clean and noisy ECG signals were considered in the evaluation process. Up to 96.6% accuracy levels were attained in this work.

A wearable sensor prototype was developed by Blasco et al.^26^ to collect ECG, PPG, and Galvanic Skin Response (GSR) signals in order to establish a multi-modal biometric system for user authentication. After filtering, each signal is divided into 2-s windows. Ninety-six coefficients are recovered using the PPG and ECG windows (sixty-four from the Fourier transform and sixty-four from the Walsh-Hadamard transform), however, four statistical features are retrieved from the GSR window. The density estimation classifier that the authors used is based on the Gaussian model, and it produced results of 0.99 for AROC and 0.02 for Equal Error Rate (EER).

ECG and audio signals were integrated by Bugdol et al.^27^ to create a behavior-based biometric system. The foundation of this system is the measurement of human responses to the stimuli. The ECG signal R-R distance between consecutive R peaks and the voice-extracted  Mel-Frequency Cepstral Coefficients (MFCCs) are used as the multi-modal system discriminant features. The system was evaluated using KNN and NN classifiers, with average accuracies of 75% and 77%, respectively. An overview of previously-published research that is closely relevant to the subject is shown in Table 1.

**Table 1:** Overview of the relevant works.

Reference	Number of Subjects	Acquisition method or database	Classifier Type	Performance Metrics	Limitations
Lee and Kwak^17^	1- 100 2- 290	1- CU-ECG DB 2- PTB-ECG DB	EECGNet-based SVM	Accuracy = 98.25%	Utilization of merely two datasets. Making use of the initial ECG templates. Additional complexity by transforming ECG signals into images.
Barros et al.^19^	1- 1500 2- 100	2018 database for PhysioNet Computing in Cardiology	RF Classifier	1-Accuracy = 92% 2-Accuracy = 95%	Just one ECG dataset is used. Making use of the initial ECG templates. Taking noise in the ECG signals into account.
Su et al.^21^	NaN	VeinECG derived from the ECG-ID and FVPolyU finger vein datasets	Discriminant Correlation Analysis (DCA)	Accuracy = 94%	Utilization of just one ECG dataset. Making use of the initial ECG templates. Taking noise in ECG signals into account.
Zhang et al.^22^	85	3 public ECG databases	Matching method	Accuracy = 97.6%	Taking noise in ECG signals into account. Making use of the initial ECG templates. Taking 4 s for authentication and 20 s for registration of a new subject.
Hammad et al.^23^	1- 25 men, and 22 women signals 2- 290 3- 65 subjects (49 males and 16 females)	1- MIT-BIH arrhythmia dataset 2- PTB dataset 3- CYBHi dataset	Feed Forward Neural Network (FFNN)	1-EER = 0.06 2-EER = 0.14 3-EER = 0.09	Disregarding noise in ECG signals.
Kim et al.^24^	89	ECG-ID database	Euclidean detection	Accuracy = 94.3%	Utilization of just one ECG dataset. Taking noise in ECG signals into account.
Zhao et al.^25^	50	Database for Physionet ECG	Convolutional Neural Network (CNN)	Accuracy = 99%	Utilization of just one dataset. Making use of the initial ECG templates. Taking noise in ECG signals into account. Additional complexity due to transforming the ECG signal into an image.
Blasco et al.^26^	25	Low-cost sensor dataset	One-class classifier with density estimation	Accuracy = 99% EER = 0.16	Utilization of just one dataset. Making use of the initial ECG templates. Taking noise in ECG signals into account.
Bugdol et al.^27^	30	Voice-ECG database	K-Nearest Neighbors (KNN) classifier	Accuracy = 89%	Utilization of just one ECG dataset. Making use of the initial ECG templates. Taking noise in ECG signals into account.

Generally, most cancellable biometric recognition systems produce acceptable results from the privacy and security perspectives. However, there are some obvious drawbacks that encourage the development of a new systems. Some of these drawbacks are listed below:

- There is a chance that the cancellable biometric recognition systems that are currently in use do not offer enough security, which puts users at the risk for identity theft, illegal access, and data breaches.
- Complex hardware or software may be needed for certain cancellable biometric recognition systems, making them challenging to set up or operate.
- The usefulness and adoption of certain cancellable biometric recognition systems are restricted, because they are neither universally scalable nor adaptable to various devices or systems.
- Users may experience inconvenience or frustration due to cancellable biometric recognition systems' inconsistent accuracy or speed.
- Usability problems might arise from unclear or difficult-to-use cancellable biometric recognition systems.

Consequently, the relatively high complexity of segmentation and classification algorithms is a feature of most of the available ECG identification systems, whether cancellable or open. The patients require an interactive system to control the basic acquisition and ECG signal encryption or deformation method in order to access the IoMT networks. Furthermore, for these tasks to be carried out automatically without the need for user intervention, a hardware implementation is necessary. The user’s only rule is to set a unique identifier that can be changed in case of hacking. That is what we will introduce in the following parts, together with the details of the proposed framework, its analysis and comments, and its superior performance compared to previous relevant studies.

## Proposed cancellable ECG recognition framework

The suggested framework for developing cancellable ECG templates is presented in this section. The ability to create cancellable templates from original ones that cannot be utilized to retrieve the original templates again is the most important feature of a cancellable biometric system. In this method, the user’s privacy is protected. The ability to modify the cancellable templates in hacking scenarios is a crucial and requested functionality. Two more essential requirements are high classification accuracy and ease of implementation.

**Figure 1:**
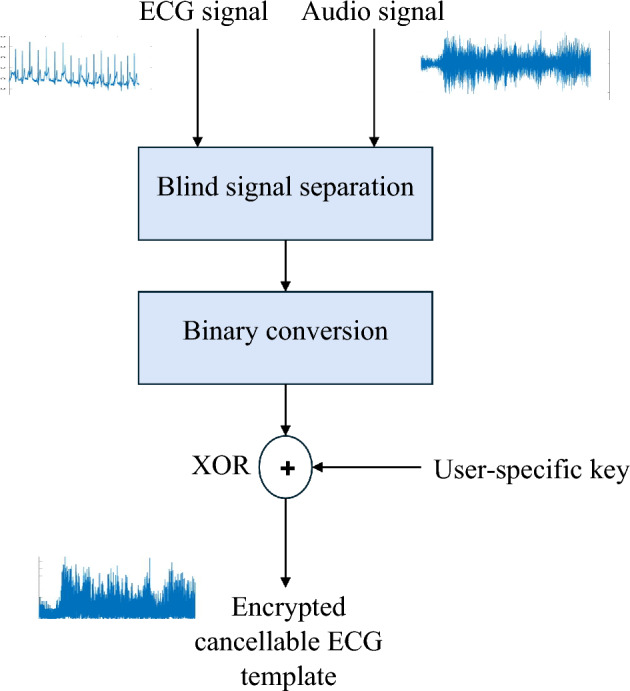
Cancellable ECG template generation steps.

The suggested framework has a hybrid design. As illustrated in Fig. 1, it is composed of a  signal deformation stage based on blind signal separation and lightweight encryption represented by binary XOR with a user-specific key. This framework aims to improve privacy by using low-cost, lightweight encryption, while rendering biometric templates non-invertible.

The 2 × 2 blind signal separation algorithm is used with two inputs, namely the ECG signal and an auxiliary audio signal. The basic idea of signal separation is to produce two signals that are uncorrelated from the two signals that have some sort of correlation. This guarantees an appropriate level of distortion to hide the significant features of the original ECG signals. Additionally, the utilization of a user-specific secret key with the same length as that of the ECG signal to be XORed with the signal supports lightweight encryption for more hiding of the signal details. The suggested framework provides secure cancellable ECG templates that can be used to access IoMT networks.

The following steps illustrate the suggested framework methodology:

1. Acquire a 1-D biometric ECG signal for the patient.
2. Acquire a 1-D audio signal for the same patient.
3. Verify that the lengths of the two signals are equal.
4. Create the updated ECG template using a blind signal separation algorithm between the ECG signal and the auxiliary audio signal.
5. Apply a binarization process to one of the two outputs of the separation process.
6. Perform XOR operation using a user-specific key with the obtained binary vector to produce the cancellable template.

System mismatch or ambient noise may have an impact on the ECG signals during the acquisition procedure. Consequently, it is recommended to remove the noise before proceeding to additional processing phases. However, in order to work on real settings, the performance of the proposed framework is tested with noisy signals at different SNRs. Additive White Gaussian Noise (AWGN) is investigated as the noise affecting the signals with an SNR of 10 dB.

The essential stage of the proposed framework is blind signal separation. It primarily addresses mixed signals, which are common in everyday life. Unwanted signals are commonly combined with signals of interest in real life. The development of blind signal separation algorithms has been spurred by this fact. The term “blind” refers to the lack of prior knowledge regarding the sources and mixed signals. Here, we use a 2 × 2 signal separation system. Its foundation is the application of output decorrelation as the criterion for signal separation. The mathematical model of the signal separation algorithm is discussed below assuming two convolutive mixtures are available^28,29^. This algorithm will be exploited with the ECG signal and the auxiliary audio signal as inputs. Our objective here is merely making use of the signal decorrelation concept to obtain distorted ECG signals that can be used as cancellable templates. 

**Figure 2:**
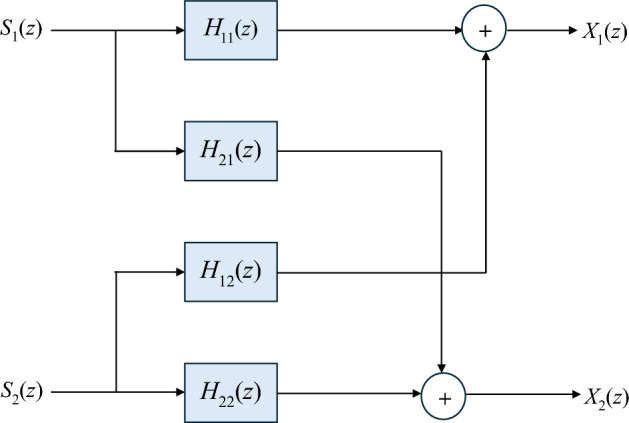
An entirely-connected 2 × 2 mixing system.

If there are two signal sources $s_{1} \left( k \right)$, $s_{2} \left( k \right)$ and two observations $x_{1} \left( k \right)$, $x_{2} \left( k \right)$ in a $2 \times 2$ Linear Time Invariant (LTI) system, it is assumed that the source signals are statistically independent with zero mean^30^. The following equations represent the observations, which are considered to be convolutive sums of the sources as seen in Fig. 2.

1 $$\begin{aligned} x_{1} \left( k \right) &= \mathop \sum \limits_{i = 0}^{p} h_{11} \left( i \right)s_{1} \left( {k - i} \right) + \mathop \sum \limits_{i = 0}^{p} h_{12} \left( i \right)s_{2} \left( {k - i} \right) \\ x_{2} \left( k \right) &= \mathop \sum \limits_{i = 0}^{p} h_{21} \left( i \right)s_{1} \left( {k - i} \right) + \mathop \sum \limits_{i = 0}^{p} h_{22} \left( i \right)s_{2} \left( {k - i} \right) \end{aligned}$$

In matrix form, we have:

2 $$\left[ {\begin{array}{*{20}c} {{\mathbf{x}}_{1} \left( k \right)} \\ {{\mathbf{x}}_{2} \left( k \right)} \\ \end{array} } \right] = \left[ {\begin{array}{*{20}c} {{\mathbf{h}}_{11}^{{T}} } & {{\mathbf{h}}_{12}^{{T}} } \\ {{\mathbf{h}}_{21}^{{T}} } & {{\mathbf{h}}_{22}^{{T}} } \\ \end{array} } \right]\left[ {\begin{array}{*{20}c} {\mathbf{s}_{1} \left( k \right)} \\ {\mathbf{s}_{2} \left( k \right)} \\ \end{array} } \right]$$

where $${\mathbf{h}}_{ij}^{T} = \left[ {h_{ij} \left( 0 \right), \ldots..,h_{ij} \left( p \right)} \right]$$.

and

3 $${\mathbf{s}}_{i}^{T} \left( k \right) = \left[ {s_{i} \left( k \right), \ldots..,s_{i} \left( {k - p} \right)} \right]$$

${h}_{ij}$ is a representation of the impulse response from source $j$ to sensor $i$, and the filter order is denoted by $p$. For simplicity, the source signals are assumed to be zero-mean and statistically independent. It is evident from Eqs. 1 and 2 that in the presence of noise, the mixtures are convolutive sums of sources. Assuming that the signals arrive at the sensors unfiltered, that is the problem is simplified by setting ${h}_{11} = {h}_{22} = 1$.

Using Eq. 1 z-transform, we obtain:

4 $$\left[ {\begin{array}{*{20}c} {X_{1} \left( z \right)} \\ {X_{2} \left( z \right)} \\ \end{array} } \right] = \left[ {\begin{array}{*{20}c} {H_{11} \left( z \right)} & {H_{12} \left( z \right)} \\ {H_{21} \left( z \right)} & {H_{22} \left( z \right)} \\ \end{array} } \right]\left[ {\begin{array}{*{20}c} {S_{1} \left( z \right)} \\ {S_{2} \left( z \right)} \\ \end{array} } \right]$$

Simplifying Eq. 4 leads to:

5 $$\left[ {\begin{array}{*{20}c} {X_{1} \left( z \right)} \\ {X_{2} \left( z \right)} \\ \end{array} } \right] = \left[ {\begin{array}{*{20}c} 1 & {H'_{12} \left( z \right)} \\ {H'_{21} \left( z \right)} & 1 \\ \end{array} } \right]\left[ {\begin{array}{*{20}c} {S'_{1} \left( z \right)} \\ {S'_{2} \left( z \right)} \\ \end{array} } \right]$$

where

$$S_{1}^{\prime } \left( z \right) = H_{11} \left( z \right) S_{1} \left( z \right)$$

6 $$S_{2}^{\prime } \left( z \right) = H_{22} \left( z \right) S_{2} \left( z \right)$$

$$H_{12}^{\prime } \left( z \right) = \frac{{H_{12} \left( z \right)}}{{H_{22} \left( z \right)}}$$

$$H_{21}^{\prime } \left( z \right) = \frac{{H_{21} \left( z \right)}}{{H_{11} \left( z \right)}}$$

$\mathbf{s}_{1} \left( k \right)$ and $\mathbf{s}_{2} \left( k \right)$ are the actual source signals, and ${\mathbf{h}}_{ij}$ are the true impulse responses of sources to sensors. $\mathbf{s'_{\it{i}}} \left( k \right)$ is then the signal as observed by the $i^{th}$ sensor. It is assumed that $H_{ii} \left( z \right) = 1$, and thus $\mathbf{s'_{\it{i}}} \left( k \right) = \mathbf{s}_{i}\left( k \right)$*,* and $\mathop{H'_{\it{ij}}} \left( z \right) = H_{{ij}} \left( z \right)$.

In the case of interest, $H_{ii} \left( z \right) = 1$, and hence Eq. 5 can be simplified to:

7 $$\left[ {\begin{array}{*{20}c} {X_{1} \left( z \right)} \\ {X_{2} \left( z \right)} \\ \end{array} } \right] = \left[ {\begin{array}{*{20}c} 1 & {H_{12} \left( z \right)} \\ {H_{21} \left( z \right)} & 1 \\ \end{array} } \right]\left[ {\begin{array}{*{20}c} {S_{1} \left( z \right)} \\ {S_{2} \left( z \right)} \\ \end{array} } \right]$$

Finding the signals $\mathbf{y}_{1} \left( k \right)$ and $\mathbf{y}_{2} \left( k \right)$ from $\mathbf{x}_{1} \left( k \right)$ and $\mathbf{x}_{2} \left( k \right)$ is the target of blind signal separation. We can presume that:

8 $$\left( {\begin{array}{*{20}c} {\mathbf{y}_{1} \left( k \right)} \\ {\mathbf{y}_{2} \left( k \right)} \\ \end{array} } \right) = \left( {\begin{array}{*{20}c} 1 & {{\mathbf{w}}_{1}^{T} } \\ {{\mathbf{w}}_{2}^{T} } & 1 \\ \end{array} } \right)\left( {\begin{array}{*{20}c} {\mathbf{x}_{1} \left( k \right)} \\ {\mathbf{x}_{2} \left( k \right)} \\ \end{array} } \right)$$

where

$${\mathbf{w}}_{i}^{T} = \left[ {w_{i} \left( 0 \right), \ldots,w_{i} \left( q \right)} \right]$$

$${\mathbf{x}}_{i}^{T} \left( k \right) = \left[ {x_{i} \left( k \right), \ldots,x_{i} \left( {k - q} \right)} \right]$$

then

9 $$\left[ {\begin{array}{*{20}c} {Y_{1} \left( z \right)} \\ {Y_{2} \left( z \right)} \\ \end{array} } \right] = \left[ {\begin{array}{*{20}c} 1 & {W_{1} \left( z \right)} \\ {W_{2} \left( z \right)} & 1 \\ \end{array} } \right]\left[ {\begin{array}{*{20}c} {X_{1} \left( z \right)} \\ {X_{2} \left( z \right)} \\ \end{array} } \right]$$

The result of substituting Eq. 7 into Eq. 9 is:

10 $$\left[ {\begin{array}{*{20}c} {Y_{1} \left( z \right)} \\ {Y_{2} \left( z \right)} \\ \end{array} } \right] = \left[ {\begin{array}{*{20}c} {1 + {W}_{1} \left( z \right)H_{21} \left( z \right)} & {{W}_{1} \left( z \right) + H_{12} \left( z \right)} \\ {{W}_{2} \left( z \right) + H_{21} \left( z \right)} & {1 + {W}_{2} \left( z \right)H_{12} \left( z \right)} \\ \end{array} } \right]\left[ {\begin{array}{*{20}c} {S_{1} \left( z \right)} \\ {S_{2} \left( z \right)} \\ \end{array} } \right]$$

Finding appropriate ${W}_{i} \left( z \right)$ such that $Y_{1} \left( z \right)$ and $Y_{2} \left( z \right)$ each contains either $S_{1} \left( z \right)$ or $S_{2} \left( z \right)$ only is the blind signal separation task.

### Iterative separation algorithm

**Figure 3:**
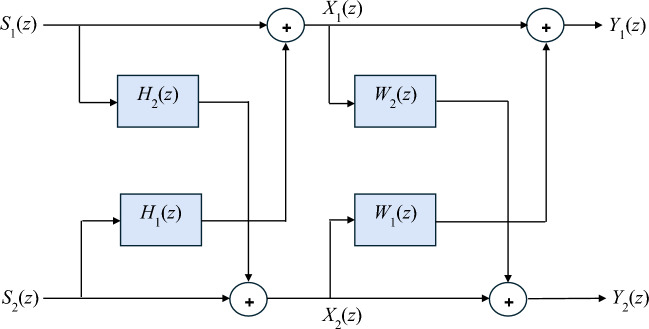
Schematic diagram of the 2 × 2 blind signal separation algorithm.

This section presents an iterative separation algorithm for the 2 × 2 convolutive system in the time domain. As shown in Fig. 3, using $q + 1$ tap Finite Impulse Response (FIR) filters, the separation algorithm minimizes the output cross-correlations for an arbitrary number of lags^30^.

Finding suitable ${W}_{1} \left( z \right)$ and ${W}_{2} \left( z \right)$ such that $Y_{1} \left( z \right)$ and $Y_{2} \left( z \right)$ each contains only either $S_{1} \left( z \right)$ or $S_{2} \left( z \right)$ is the solution for the problem, according to Eq. 9. Given stationary, zero-mean, independent random signals $\mathbf{s}_{1} \left( k \right)$ and $\mathbf{s}_{2} \left( k \right)$, their cross-correlation is equal to zero, which means that:

11 $$\mathbf{r}_{{s_{1} s_{2} }} \left( l \right) = E\left[ {\mathbf{s}_{1} \left( k \right)\mathbf{s}_{2} \left( {k + l} \right)} ^{T}\right] = 0 \,\forall \,l$$

The cross-correlation of $\mathbf{y}_{1} \left( k \right)$ and $\mathbf{y}_{2} \left( k \right)$ should also be zero if $\mathbf{y}_{1} \left( k \right)$ and $\mathbf{y}_{2} \left( k \right)$ each includes components of either $\mathbf{s}_{1} \left( k \right)$ or $\mathbf{s}_{2} \left( k \right)$, only. Hence,

12 $$\mathbf{r}_{{y_{1} y_{2} }} \left( l \right) = E\left[ {\mathbf{y}_{1} \left( k \right)\mathbf{y}_{2} \left( {k + l} \right)} ^{T}\right] = 0 \,\forall \,l{ }$$

Substituting from Eq. 8 into Eq. 12 yields:

13 $$\mathbf{r}_{{y_{1} y_{2} }} \left( l \right) = E\left[ {(\mathbf{x}_{1} \left( k \right) + {\mathbf{w}}_{1}^{T} \mathbf{x}_{2} \left( k \right))(\mathbf{x}_{2} \left( {k + l} \right) + {\mathbf{w}}_{2}^{T} \mathbf{x}_{1} \left( {k + 1} \right))} ^{T}\right]{ }$$

Denote that $\mathbf{r}_{{x_{1} x_{2} }} \left( l \right) = E[\mathbf{x}_{1} \left( k \right)\mathbf{x}_{2} \left( {k + l} \right)^{T}]$. Equation 13 becomes:

14 $$\mathbf{r}_{{y_{1} y_{2} }} \left( l \right) = \mathbf{r}_{{x_{1} x_{2} }} \left( l \right) + {\mathbf{w}}_{1}^{T} \left[ {\begin{array}{*{20}c} {r_{{x_{2} x_{2} }} \left( l \right)} \\ \vdots \\ {r_{{x_{2} x_{2} }} \left( {l + q} \right)} \\ \end{array} } \right] + {\mathbf{w}}_{2}^{T} \left[ {\begin{array}{*{20}c} {r_{{x_{1} x_{1} }} \left( l \right)} \\ \vdots \\ {r_{{x_{1} x_{1} }} \left( {l + q} \right)} \\ \end{array} } \right] + {\mathbf{w}}_{1}^{T} {\varvec{r}}_{{x_{2} x_{1} }} \left( l \right){\mathbf{w}}_{2}$$

where ${\mathbf{r}}_{{x_{2} x_{1} }} \left( l \right) = E[\mathbf{x}_{2} \left( k \right)\mathbf{x}_{1} \left( {k + l} \right)^{T}]$ is a $\left( {q + 1} \right) \times \left( {q + 1} \right)$ matrix representing the cross-correlation of $\mathbf{x}_{2}$ and $\mathbf{x}_{1}$.

The sum of the squares of the cross-correlation elements determines the cost function *C* as:

15 $$C = \mathop \sum \limits_{{l = l_{1} }}^{{l_{2} }} r^{2}_{{y_{1} y_{2} }} \left( l \right)$$

where $C$ can also be written as in Eq. 16, and $l_{1}$ and $l_{2}$ are selected cross-correlation lags.

16 $$C = {\mathbf{r}}_{{y_{1} y_{2} }}^{T} {\mathbf{r}}_{{y_{1} y_{2} }}$$

where

17 $$\mathbf{r}_{{{y}_{1} {y}_{2} }} = \left[ {r_{{{y}_{1} {y}_{2} }} \left( {l_{1 } } \right), \ldots,\user2{ }r_{{{y}_{1} {y}_{2} }} \left( {l_{2} } \right)} \right]^{{T}}$$

In matrix notation, Eq. 17 can be represented as:

18 $${\mathbf{r}}_{{y_{1} y_{2} }} = {\mathbf{r}}_{{x_{1} x_{2} }} + \left[ {{\mathbf{Q}}_{{x_{2} x_{2} }}^{ + } } \right]^{T} {\mathbf{w}}_{1} + \left[ {{\mathbf{Q}}_{{x_{1} x_{1} }}^{ - } } \right]^{T} {\mathbf{w}}_{2} + {\mathbf{r}}_{{x_{2} x_{1} }}^{T} {\mathbf{A}}\left( {{\mathbf{w}}_{2} } \right){\mathbf{w}}_{1}$$

or

19 $${\mathbf{r}}_{{y_{1} y_{2} }} = {\mathbf{r}}_{{x_{1} x_{2} }} + \left[ {{\mathbf{Q}}_{{x_{2} x_{2} }}^{ + } } \right]^{T} {\mathbf{w}}_{1} + \left[ {{\mathbf{Q}}_{{x_{1} x_{1} }}^{ - } } \right]^{T} {\mathbf{w}}_{2} + {\mathbf{r}}_{{x_{1} x_{2} }}^{T} {\mathbf{A}}\left( {{\mathbf{w}}_{1} } \right){\mathbf{w}}_{2}$$

where ${\mathbf{Q}}_{{x_{2} x_{2} }}^{ + }$ and ${\mathbf{Q}}_{{x_{1} x_{1} }}^{ - }$ are $\left( {q + 1} \right) \times \left( {l_{2} - l_{1} + 1} \right)$ matrices, ${\mathbf{r}}_{{x_{2} x_{1} }}$ is a $\left( {2q + 1} \right) \times \left( {l_{2} - l_{1} + 1} \right)$ matrix. These are sample estimates for the correlation of ${\mathbf{x}}_{1}$ and ${\mathbf{x}}_{2}$. ${\mathbf{A}}\left( {{\mathbf{w}}_{1} } \right)$ and ${\mathbf{A}}\left( {{\mathbf{w}}_{2} } \right)$ are $\left( {2q + 1} \right) \times \left( {q + 1} \right)$ matrices, which contain ${\mathbf{w}}_{1}$ and ${\mathbf{w}}_{2}$, respectively. To estimate ${\mathbf{w}}_{1}$ and ${\mathbf{w}}_{2}$, $C$ is differentiated, such that:

20 $$\frac{\partial C}{{\partial \mathbf{w}_{i} }} = \left[ {0, \ldots,0} \right]^{T}, i = 1,2$$

Let

21 $$\psi_{1} = \left( {\left[ {{\mathbf{Q}}_{{x_{2} x_{2} }}^{ + } } \right]^{T} + {\mathbf{r}}_{{x_{2} x_{1} }}^{T} {\mathbf{A}}\left( {{\mathbf{w}}_{2} } \right)} \right)$$

$$\psi_{2} = \left( {\left[ {{\mathbf{Q}}_{{x_{1} x_{1} }}^{ - } } \right]^{T} + {\mathbf{r}}_{{x_{1} x_{2} }}^{T} {\mathbf{A}}\left( {{\mathbf{w}}_{1} } \right)} \right)$$

Substituting Eqs. 14 and 21 into Eqs. 18 and 19 gives:

22 $$\mathbf{r}_{{y_{1} y_{2} }} = \mathbf{r}_{{x_{1} x_{2} }} + \psi_{1} {\mathbf{w}}_{1} + \left[ {{\mathbf{Q}}_{{x_{1} x_{1} }}^{ - } } \right]^{T} {\mathbf{w}}_{2}$$

or

23 $$\mathbf{r}_{{y_{1} y_{2} }} = \mathbf{r}_{{x_{1} x_{2} }} + \psi_{2} {\mathbf{w}}_{2} + \left[ {{\mathbf{Q}}_{{x_{2} x_{2} }}^{ + } } \right]^{T} {\mathbf{w}}_{1}$$

From Eqs. 22 and 23 , we obtain:

24 $${\mathbf{w}}_{1} = - \left( {\psi_{1}^{T} \psi_{1} } \right)^{ - 1} \psi_{1}^{T} \left( {\mathbf{r}_{{x_{1} x_{2} }} + \left[ {{\mathbf{Q}}_{{x_{1} x_{1} }}^{ - } } \right]^{T} {\mathbf{w}}_{2} } \right)$$

$${\mathbf{w}}_{2} = - \left( {\psi_{2}^{T} \psi_{2} } \right)^{ - 1} \psi_{2}^{T} \left( {\mathbf{r}_{{x_{1} x_{2} }} + \left[ {{\mathbf{Q}}_{{x_{2} x_{2} }}^{ + } } \right]^{T} {\mathbf{w}}_{1} } \right)$$

**Figure 4:**
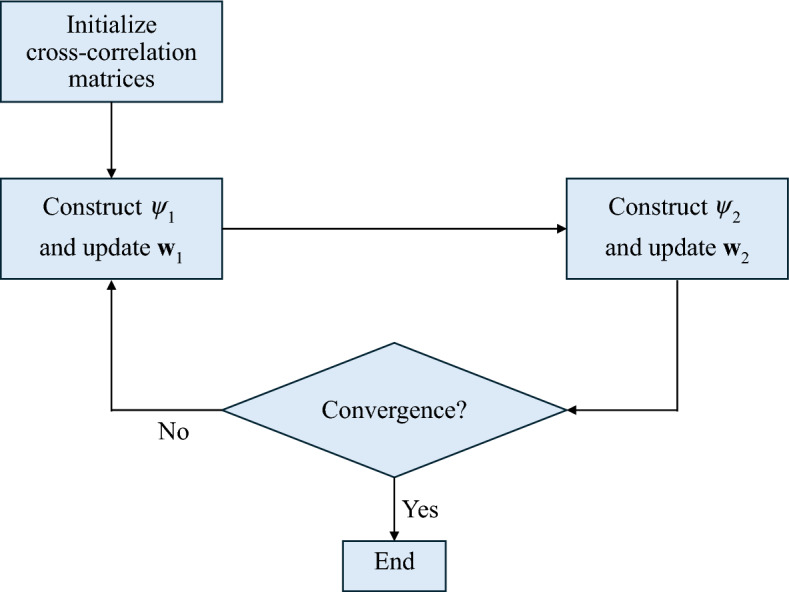
Flowchart of weight optimization for blind signal separation.

When the rate of change of the derivative in Eq. 20 is smaller than a pre-set threshold, such as 0.01%, convergence is reached, and ${\mathbf{w}}_{1}$ and ${\mathbf{w}}_{2}$ can be determined by iterating between the two equations. We then get a set of outputs, $\mathbf{y}_{1} \left( k \right)$ and $\mathbf{y}_{2} \left( k \right)$, by estimating ${\mathbf{w}}_{1}$ and ${\mathbf{w}}_{2}$. Only $\mathbf{s}_{1} \left( k \right)$ or $\mathbf{s}_{2} \left( k \right)$ is present in each output^30^. Fig. 4 shows the weight optimization process.

### Algorithm steps

1. The cross-correlation matrices ${\mathbf{Q}}_{{x_{1} x_{1} }}^{ - }$ and ${\mathbf{Q}}_{{x_{2} x_{2} }}^{ + }$ are initialized.
2. The matrices $\psi_{1}$ and $\psi_{2}$ are constructed.
3. The weights ${\mathbf{w}}_{1}$ and ${\mathbf{w}}_{2}$ are updated.
4. Convergence is checked.
5. Iterative update of weights ${\mathbf{w}}_{1}$ and ${\mathbf{w}}_{2}$ continues until the cost function *C* is minimized and convergence occurs.
6. The weights ${\mathbf{w}}_{1}$ and ${\mathbf{w}}_{2}$ at which convergence occurs are selected as the optimum weights.
7. Since optimum weights ${\mathbf{w}}_{1}$ and ${\mathbf{w}}_{2}$ are obtained, the outputs $\mathbf{y}_{1} \left( k \right)$ and $\mathbf{y}_{2} \left( k \right)$ can be obtained.
8. The cancellable template is selected as either $\mathbf{y}_{1} \left( k \right)$ or $\mathbf{y}_{2} \left( k \right).$

## Experiments

### ECG datasets

In this work, two public and accessible ECG datasets were used to assess the efficacy of the proposed cancellable biometric recognition framework based on ECG signals: ECG-ID^31–33^, and MIT-BIH^34–37^. Using a single-lead ECG sensor, 310 ECG records for 90 people (46 women and 44 men) have been acquired to constitute the ECG-ID dataset. Every record is 20-s long and has a 12-bit resolution with a sampling rate of 500 Hz. A few demographic details, including age, gender, and the recording date, are also included in the dataset. There are 48 two-channel ECG recordings in the MIT-BIH dataset, each lasting for 30 min. The recordings are for 47 different persons with a 11-bit resolution and a sampling rate of 360 Hz for each channel.

### Steps of the proposed framework for IoMT network access

**Figure 5:**
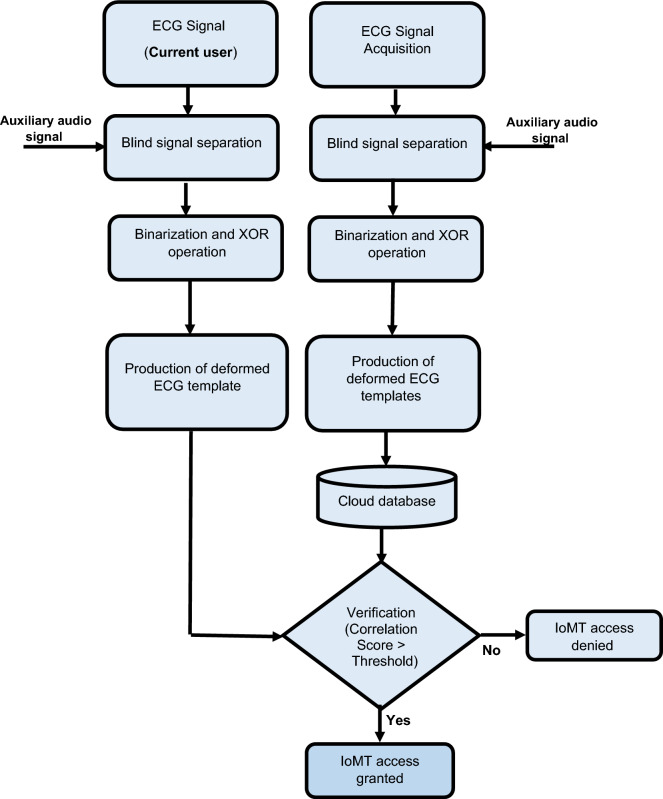
Flowchart of the proposed framework for IoMT networks access.

Figure 5 illustrates the proposed IoMT network access framework based on ECG signals. Its main steps are summarized as follows:

- Acquire of ECG signals.
- Create distorted ECG templates through blind signal separation with an auxiliary audio signal for the same person and XOR encryption operation. These templates are either stored in a database or used for authentication.
- Use a correlation metric to verify user identity.

#### ECG signal acquisition

The first step is to obtain the required ECG signal using non-invasive electrodes.

#### Preprocessing of ECG signals

System mismatch or ambient noise may have an impact on the ECG signals during acquisition. A digital notch filter can be implemented to eliminate power line interference.

#### Production of cancellable ECG templates

Non-invertible cancellable ECG templates are generated based on blind signal separation with the help of an auxiliary audio signal, which induces some sort of distortion into the signals. We also use lightweight encryption with XOR operation and user-specific keys to enhance the level of security.

#### Classification and verification processes

Based on the correlation score between the query and the biometric templates stored in the database, matching scores are obtained. The matching sores are used for user verification. A significant degree of similarity between two templates is indicated by a high correlation score. We first generate genuine and imposter correlation distributions, which allows us to set a threshold for discrimination and determine the EER value. High security is indicated by the low EER. The similarity correlation sore between a new query template and the stored ones is calculated during the verification stage and compared to the threshold for decision making. A correlation score higher than the threshold means a matching case.

### Evaluation and results

This section presents the evaluation of the proposed framework using two essential metrics. The first metric is the correlation score, which determines the degree of similarity between a new cancellable template and the ones stored in the database, according to the following relation:

25 $$R_{xy} = \frac{{C_{v} \left( {x,y} \right)}}{{\sigma_{x} \sigma_{y} }}$$

where $C_{v}$ is the covariance between the database-stored cancellable ECG template, represented by $x$, and the new cancellable template during the authentication step, represented by $y$. $\sigma_{x}$ and $\sigma_{y}$ are the standard deviations of the templates.

The second metric is the AROC. It represents the effectiveness of the authentication system^38–40^. The ROC curve is obtained by plotting the False Positive Rate (FPR) versus the True Positive Rate (TPR). The TPR is the system sensitivity indicator that shows the likelihood of correctly-classified states. The probability of incorrectly rejecting states is measured by the FPR. The following formulas are used to represent TPR and FPR^40^:

26 $$TPR = \frac{{True{ }\;positives}}{{Total\;{ }number{ }\;of\;{ }positives}}$$

27 $$FPR = \frac{{False{ }\;positives}}{{Total\;{ }number\;{ }of{ }\;negatives}}$$

**Figure 6:**
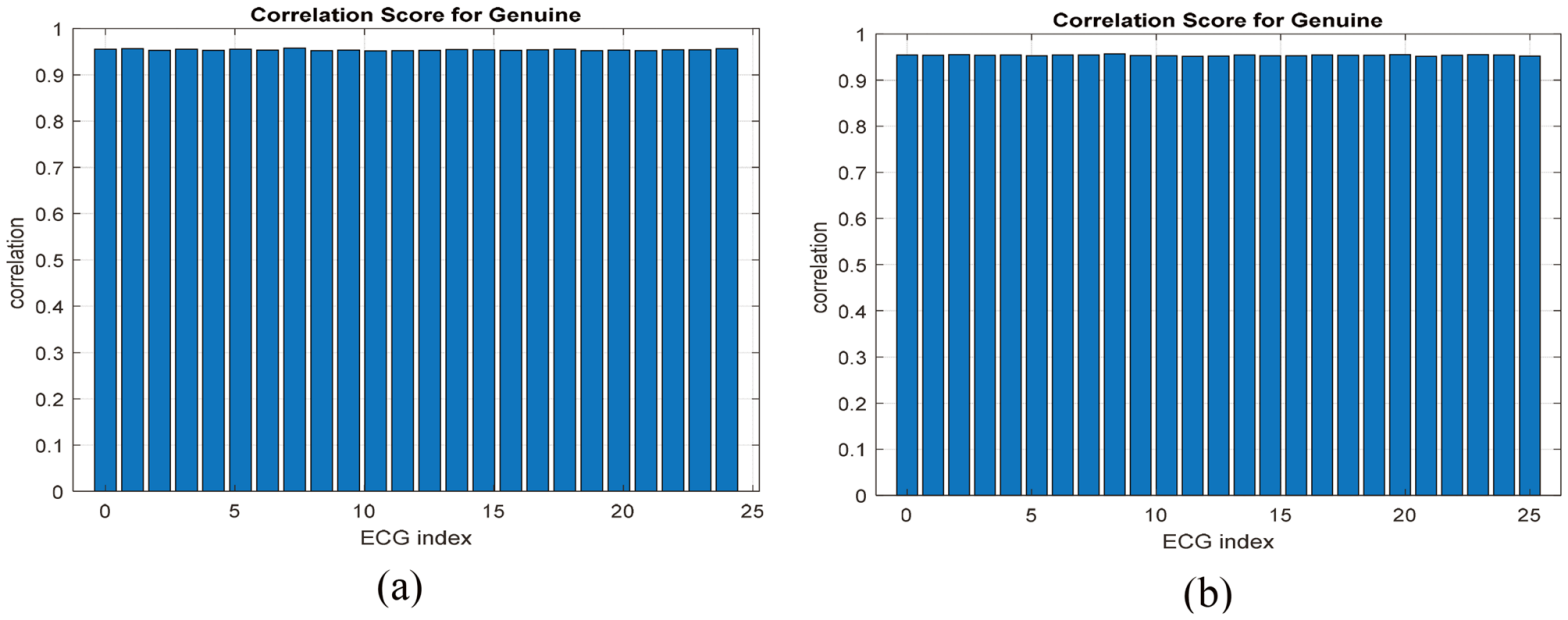
Correlations of approved ECG biometrics: (**a**) for the ECG-ID dataset and (**b**) for the MIT-BIH dataset.

**Figure 7:**
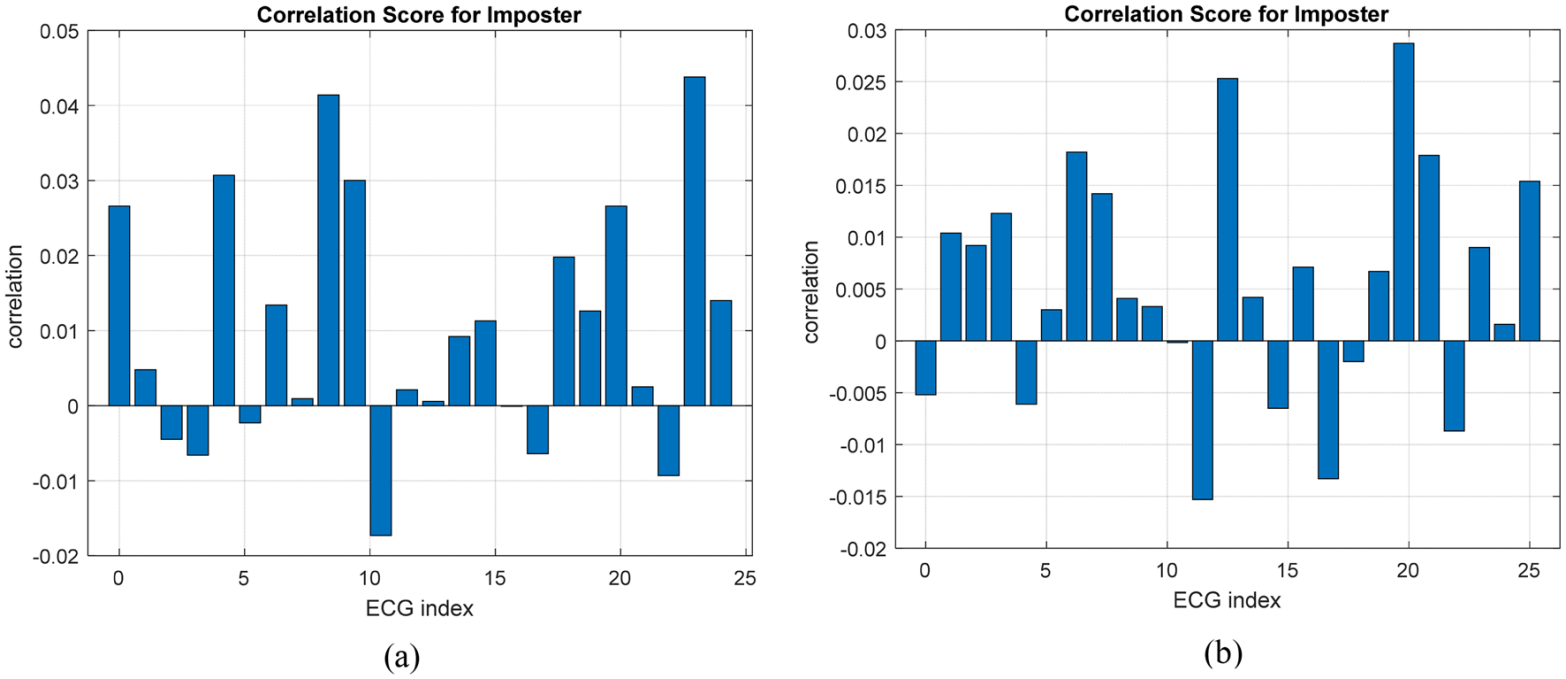
Correlation scores for imposters: (**a**) for the ECG-ID dataset and (**b**) for the MIT-BIH dataset.

The correlation scores for approved encrypted biometrics for genuine users are displayed in Fig. 6. Comparably, the correlation scores for impostor biometrics are shown in Fig. 7. The results show that all correlation values for genuine users are larger than 0.95, whereas those for imposters are less than 0.05. As a result, it is easy to set a threshold value in the range of 0.05 to 0.95 to distinguish between biometrics of genuine users and those of imposters. This ensures that the suggested framework has a high level of security.

**Figure 8:**
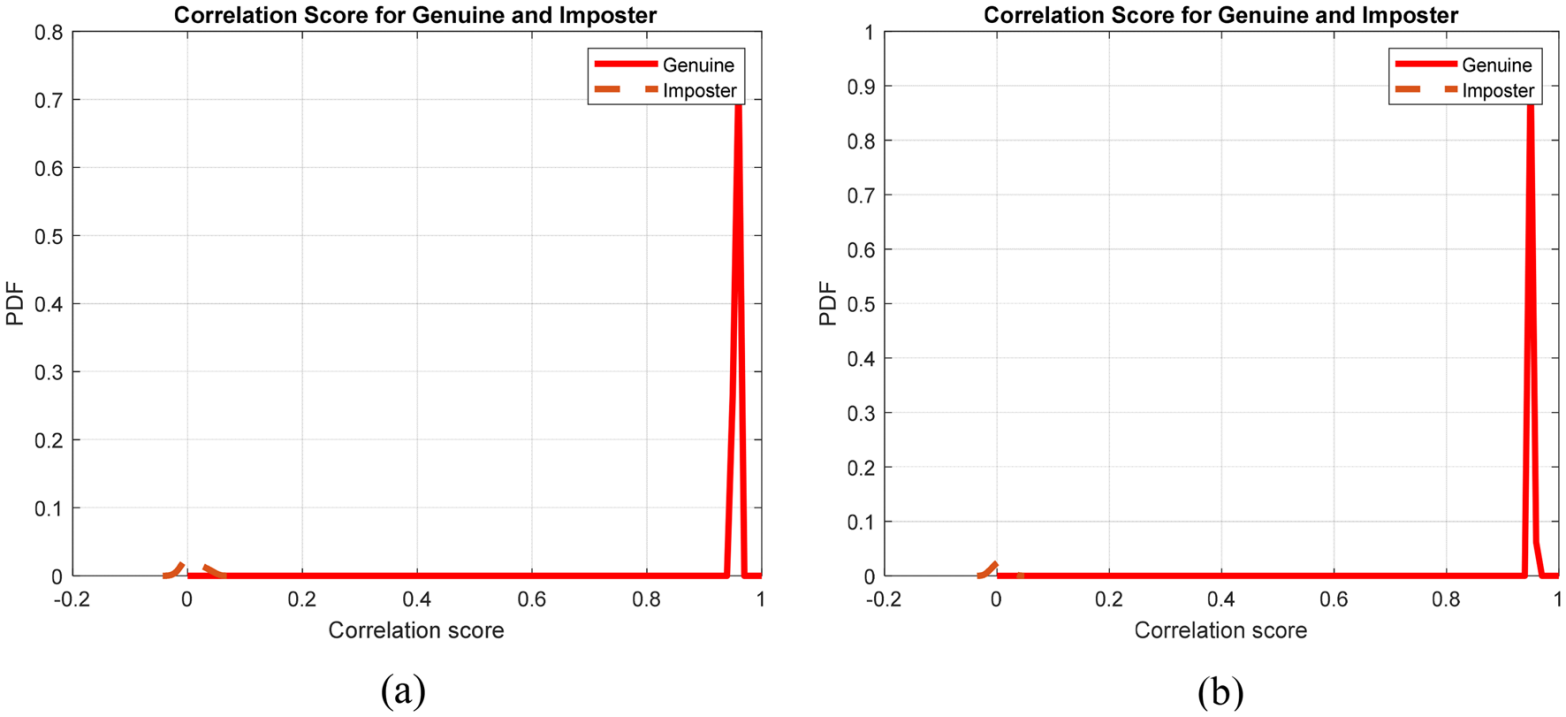
Genuine and imposter distributions of the proposed cancellable ECG recognition framework: (**a**) for the ECG-ID dataset, and (**b**) for the MIT-BIH dataset.

**Figure 9:**
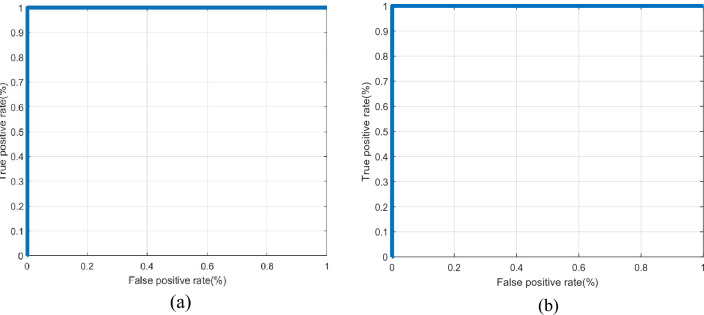
Receiver operating characteristic (ROC) curves for the proposed cancellable biometric recognition framework: (**a**) for the ECG-ID dataset and (**b**) for the MIT-BIH dataset.

**Figure 10:**
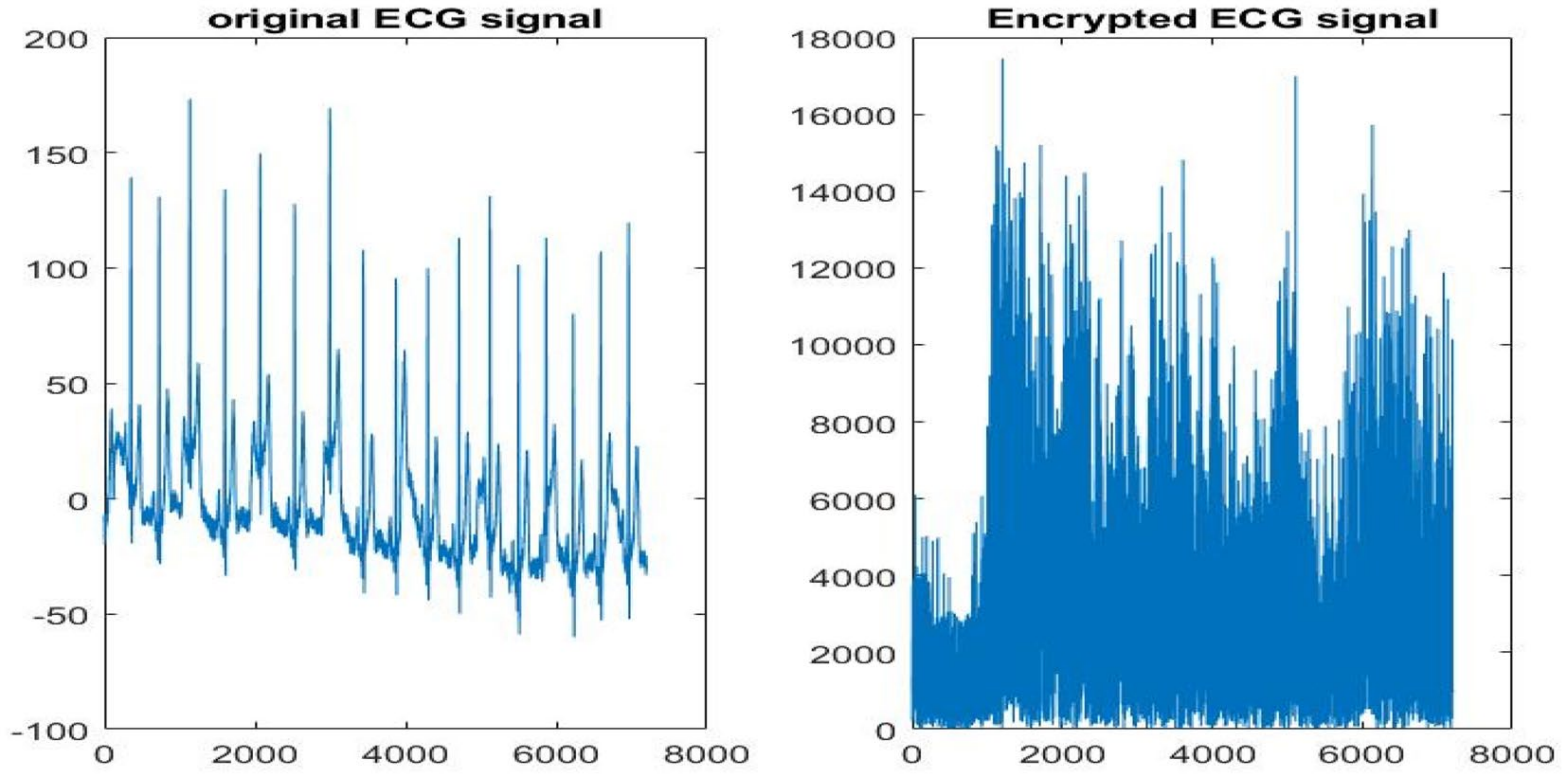
Original and cancellable ECG signals.

To give more credibility to the results, Fig. 8 displays the genuine and imposter probability distributions, ensuring low EER values. In addition, Fig. 9 shows the ROC curves of the proposed framework on the two datasets revealing high AROC values. Moreover, the original and cancellable ECG templates are shown in Fig. 10, ensuring dissimilarity between the templates.

A comparison between the proposed cancellable biometric recognition framework and other systems is presented in Table 2. This table shows the performance of the proposed framework at an SNR of 10 dB. The outcomes show that the suggested framework outperforms the other systems. The strength of the proposed framework is reflected by the high accuracy value of 99.96% at the SNR of 10 dB.

**Table 2:** Comparison of the proposed framework with other previous systems.

Work	Dataset	EER	Accuracy (%)
Barros et al.^19^	PhysioNet	N/A	92
Su et al.^21^	ECG-ID	0.144	75.71
Zhang et al.^22^	ptbdb, mitdb, nsrdb	1.57	97.6
Hammad et al.^23^	MIT-BIH	6	N/A
Kim et al.^24^	ECG-ID	2.6	94.3
Zhao et al.^25^	ECG-ID	5.68	96.6
Blasco et al.^26^	Low-cost sensors biometrics	2	99
Proposed framework at SNR = 10 dB	ECG-ID	0.134	99.96
	MIT-BIH	0.4	99.96

## Conclusions and future work

This research presented a new cancellable biometric recognition framework that utilizes unique ECG signals to secure the IoMT network access process. The methodology adopted herein combines blind signal separation with lightweight encryption. It guarantees balancing between security demands and operational efficiency. Such a balance is critical in healthcare contexts, where the immediacy of access to medical data must not compromise the integrity and confidentiality of patient information. Moreover, the practicality of our solution, characterized by its adaptability to mobile hardware, paves the way for broader adoption and integration into existing IoMT ecosystems. It underscores the potential for cancellable biometric frameworks to evolve beyond traditional security mechanisms, offering a dual advantage of enhanced security and user-centric design. This research, therefore, not only addresses current security challenges within IoMT networks but also anticipates the future needs of the new healthcare landscape. In doing so, it invites a paradigm shift in how security is conceptualized and implemented in medical technology, advocating for solutions that are both technologically advanced and deeply attuned to the human aspects of healthcare delivery. Our findings demonstrate that this framework offers a high degree of security, evidenced by low EER and high AROC values. These promising outcomes give a rich avenue for exploration, particularly in the development of more sophisticated algorithms and the exploration of other biometric modalities, to further refine and enhance the security and usability of IoMT systems. As we look forward, it is imperative that the research community continues to innovate and collaborate in developing security solutions that not only protect but also empower patients and healthcare providers in the digital age.

The limitations of this work can be summarized as follows:

- Limited resilience to diverse cyber threats due to the specific use of a 2 × 2 separation model and lightweight encryption.
- ECG-ID and MIT-BIH datasets may not fully capture the wide range of patient demographics and ECG signal variations.
- Limited ability to adapt to rapidly evolving cyber threats, which may affect long-term effectiveness.
- Compatibility and implementation challenges, when integrating with current IoMT infrastructures.
- Issues regarding user acceptance and usability, particularly in urgent care scenarios.

Thus, future investigations will aim to further refine and expand the capabilities of our framework, with a particular focus on:

- Exploring additional biometric modalities and advanced machine learning algorithms to enhance accuracy.
- Investigating sophisticated signal processing and encryption techniques to fortify against emerging threats.
- Assessing the framework applicability to broader healthcare scenarios, including patient identification and medical record management.
- Optimizing the system for real-time processing to support instant authorization and authentication in IoMT devices.
- Enhancing the framework resistance to environmental variations and sophisticated cyberattacks.

## Data Availability

All information is available from the corresponding author upon request.
